# Convergence of Multiple Stimuli to a Single Gate in TREK1 and TRAAK Potassium Channels

**DOI:** 10.3389/fphar.2021.755826

**Published:** 2021-09-30

**Authors:** Frank S Choveau, Ismail Ben Soussia, Delphine Bichet, Chatelain C. Franck, Sylvain Feliciangeli, Florian Lesage

**Affiliations:** Université Côte D’Azur, INSERM, CNRS, Institut de Pharmacologie Moléculaire et Cellulaire, LabEx ICST, Valbonne, France

**Keywords:** two-pore domain potassium channels, pore gating, openers, allostery, extracellular pH

## Abstract

Inhibitory potassium channels of the TREK1/TRAAK family are integrators of multiple stimuli, including temperature, membrane stretch, polyunsaturated fatty acids and pH. How these signals affect the gating of these channels is the subject of intense research. We have previously identified a cytoplasmic domain, pCt, which plays a major role in controlling channel activity. Here, we use pharmacology to show that the effects of pCt, arachidonic acid, and extracellular pH converge to the same gate within the channel. Using a state-dependent inhibitor, fluoxetine, as well as natural and synthetic openers, we provide further evidence that the “up” and “down” conformations identified by crystallography do not correspond to open and closed states of these channels.

## Introduction

TWIK-related K^+^ channels TREK1, TREK2 and TRAAK are two-pore-domain potassium (K_2P_) channels (Enyedi and Czirjak, 2010; Feliciangeli et al., 2015). Active as homo or heterodimers (Blin et al., 2016; Lengyel et al., 2016; Levitz et al., 2016), they play important roles in the nervous system, regulating cognitive function, pain perception, neuroprotection as well as anesthesia (Heurteaux et al., 2004; Noel et al., 2011; Wang et al., 2020). They produce inhibitory background currents that are sensitive to a wide range of physical and biological stimuli including mechanical forces, temperature, pH and lipids (Maingret et al., 1999; Maingret et al., 2000a; Maingret et al., 2000b; Honore et al., 2002; Sandoz et al., 2009). They are sensitive to antidepressants and volatile anesthetics (Patel et al., 1999; Heurteaux et al., 2006). These stimuli converge on a gate that involves the selectivity filter (SF) of these channels (Bagriantsev et al., 2011). The use of quaternary ammonium (QA) ions demonstrated that unlike most K^+^ channels, in a closed state, TREK1 has no inner gate preventing the binding of QA ions deep within the pore just below SF (Piechotta et al., 2011). Intracellular acidification (Sandoz et al., 2009; Piechotta et al., 2011), mechanical forces (Bagriantsev et al., 2011) or binding of the cytoplasmic protein AKAP150 (Sandoz et al., 2006) all act indirectly on the SF gate through the fourth membrane-spanning segment (M4) and the cytoplasmic C-ter of the channel (Bagriantsev et al., 2012). TREK1, TREK2 and TRAAK are also sensitive to the extracellular pH (Sandoz et al., 2009). A single extracellular residue, H126 in TREK1 and H151 in TREK2, is involved in proton sensing. TREK1 and TRAAK are inhibited by acidification whereas TREK2 is activated. This differential effect of acidification involves charged residues close to M4 (Sandoz et al., 2009). Structural modeling and site-directed mutagenesis suggested that attraction or repulsion between the protonated side chain of histidine and these closely located negatively or positively charged residues control the gating of these channels.

The recent crystallographic 3-D structures of these channels have shown that TREK/TRAAK channels can adopt two main conformational states which show differences in the positions of the membrane-spanning segments M2 and M4, and described as the “up” and “down” conformations (Brohawn et al., 2012; Lolicato et al., 2014). Although initially attributed to open and closed states, both conformations are in fact associated with channel activity. More recently, we showed that a cytoplasmic domain, immediately following M4 (the proximal C-ter domain, named pCt), exerts opposite effects in TREK1 and TRAAK (Soussia et al., 2018). In basal conditions, pCt favors activity in TREK1 whereas it impairs activity in TRAAK. Using the conformation-dependent binding of the antidepressant fluoxetine, we gathered data suggesting that TREK1 and TRAAK conformations at rest are different, “down” for TREK1 and “up” for TRAAK, and under the influence of pCt. The differential regulation of TREK1 and TRAAK is related to a phosphatidylinositol-4, 5-bisphosphate (PIP_2_)-binding site (R329, R330, R331) present within the pCt of TREK1 and absent in TRAAK. The binding of PIP_2_ to this site results in a stabilization of the conductive conformation of TREK1 (Soussia et al., 2018). Uncoupling pCt and M4 by introducing glycine residues at the junction of these domains prevents the activating property of TREK1 pCt and the inhibitory effect of TRAAK pCt, suggesting a physical coupling between pCt and the SF gate of the channel.

Despite all these data related to the 3D-structures and regulations of these channels, the nature of the conformational changes that control the activity of these channels remains an open question. Here, by using domain swapping and the conformation-dependent binding of fluoxetine, we evaluated how pCt affects the sensitivity of TREK1 and TRAAK to external pH and openers including ML67, BL1249, and halothane, a volatile anesthetic.

## Material and Methods

**Molecular biology** - Human TREK1 (KCNK2, GenBank accession number AAH69462.1) and TRAAK (KCNK4, NCBI Reference Sequence: NP_201, 567.1) were cloned into pIRES2-eGFP vector (Clontech). All the chimeras were obtained by overlapping PCR and inserted into the same vector. Substitution of I292G293D294 in TREK1 and I253G254N255 in TRAAK by a repeat of glycines was performed by PCR using Pfu Turbo DNA polymerase (Agilent). All the constructs were verified by DNA sequencing. The exchange of pCt between TREK1 and TRAAK to generate TRAAKpCt_TREK1_ and TRAAKpCt_TREK1_ has been described elsewhere (Soussia et al., 2018).

**Cell culture and transfection -** HEK cells were grown in 100-mm tissue-culture dishes (Falcon, Franklin Lakes, NJ) in Dulbecco’s modified Eagle’s medium (Gibco, Life Technologies, Saint Aubin, France) supplemented with 10% fetal calf serum (Hyclone, Thermo Fisher Scientific GMBH, Ulm, Germany) and 1% penicillin-streptomycin (Gibco, Life Technologies, Saint Aubin, France) in a humidified incubator at 37 C (5% CO2). For expression and electrophysiology of WT and mutant channels, 0.8 µg of plasmid was transfected using JET PEI (Polyplus transfection) according to the manufacturer’s instructions. Cells were plated onto 35 mm dishes 24 h before transfection, and experiments were performed over the following 1–2 days.

**Electrophysiology -** Pipettes were pulled from haematocrit-capillaries (Hirschmann Laborgeraete, Germany) using a vertical puller (PC-10, Narishige International, London, United Kingdom), and had resistances of 2–4 MΩ when filled with internal solution and measured in standard bath solution. Whole cell membrane currents were measured and filtered at 3 kHz by a RK 400 patch clamp amplifier (Bio-Logic Science Instruments), and digitized at 10 kHz using a 12-bit analog-to-digital converter Digidata-1322 (Axon Instrument, Sunnyvale, CA, United States of America). Recordings were done using Clampex 8.2 software (Axon Instrument). The external solution used to record TREK1 and TRAAK currents in HEK cells contained (in mM): 140 NaCl, 10 TE A-Cl, 5 KCl, three MgCl_2_, one CaCl_2_, 10 HEPES, pH 7.4 with NaOH. The pipette solution contained (in mM): 155 KCl, three MgCl_2_, five EGTA, 10 HEPES, pH 7.2 with KOH. Cells were placed in 35 mm dishes through which solution flowed at 1–2 ml/min. Inflow to the dish was by gravity from several reservoirs, selectable by activation of solenoid valves (Warner Scientific). Bath solution exchange was essentially complete by < 30 s. Experiments were performed at room temperature. Cell populations were compared using Kruskall Wallis, Mann-Whitney test or Two-way ANOVA. The data are given as the mean ± SEM.

## Results

### Openers and pCt Have Allosteric Effects on the Channel Activity of TREK1 and TRAAK

**FIGURE 1:**
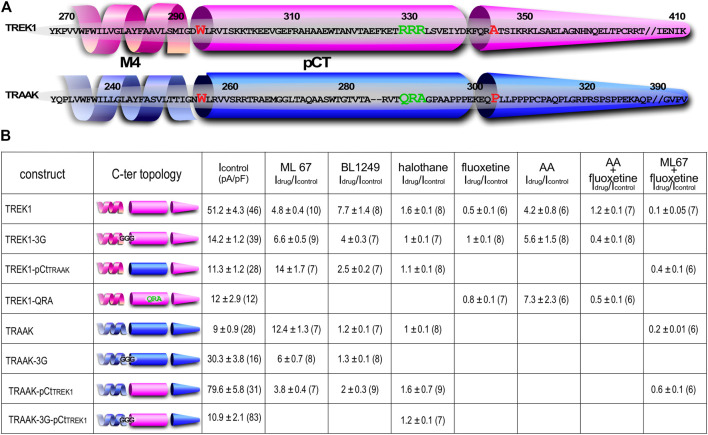
Sequence alignment and Idrug/Icontrol ratios. **(A)** Alignment of human TREK1 and TRAAK (M4: membrane-spanning segment 4, pCt: proximal C-ter domain). The residues (IGD for TREK1 and IGN of TRAAK) substituted by three glycines in TREK1-3G and TRAAK-3G mutants are shown. The residues flanking TREK1 pCt (W295 and A343) and TRAAK pCt (W256 and P302) are in red. The residues forming a PIP_2_ binding site in TREK1 (RRR), and the analogous residues (QRA) in TRAAK, are in green. This triplet R229-R230-R231 is substituted by QRA in TREK1-QRA. **(B)** I_drug_/I_control_ ratios of the corresponding channels. I_drug_ and I_control_ are the currents measured at 0 mV (pA/pF) in the presence and absence of drug as specified.

To study the impact of pCt on the activating effects of openers, we expressed chimeric channels in which pCt is swapped between TREK1 (residues W295 to A343) and TRAAK (residues W256 to P302), to give TREK1pCt_TRAAK_ and TRAAKpCt_TREK1_ (Figure 1). To physically uncouple the M4 and pCt domains in these channels, residues 292 to 294 in TREK1 were replaced by three glycines to produce TREK1-3G and residues 253 to 255 in TRAAK to produce TRAAK-3G (Figure 1).

**FIGURE 2:**
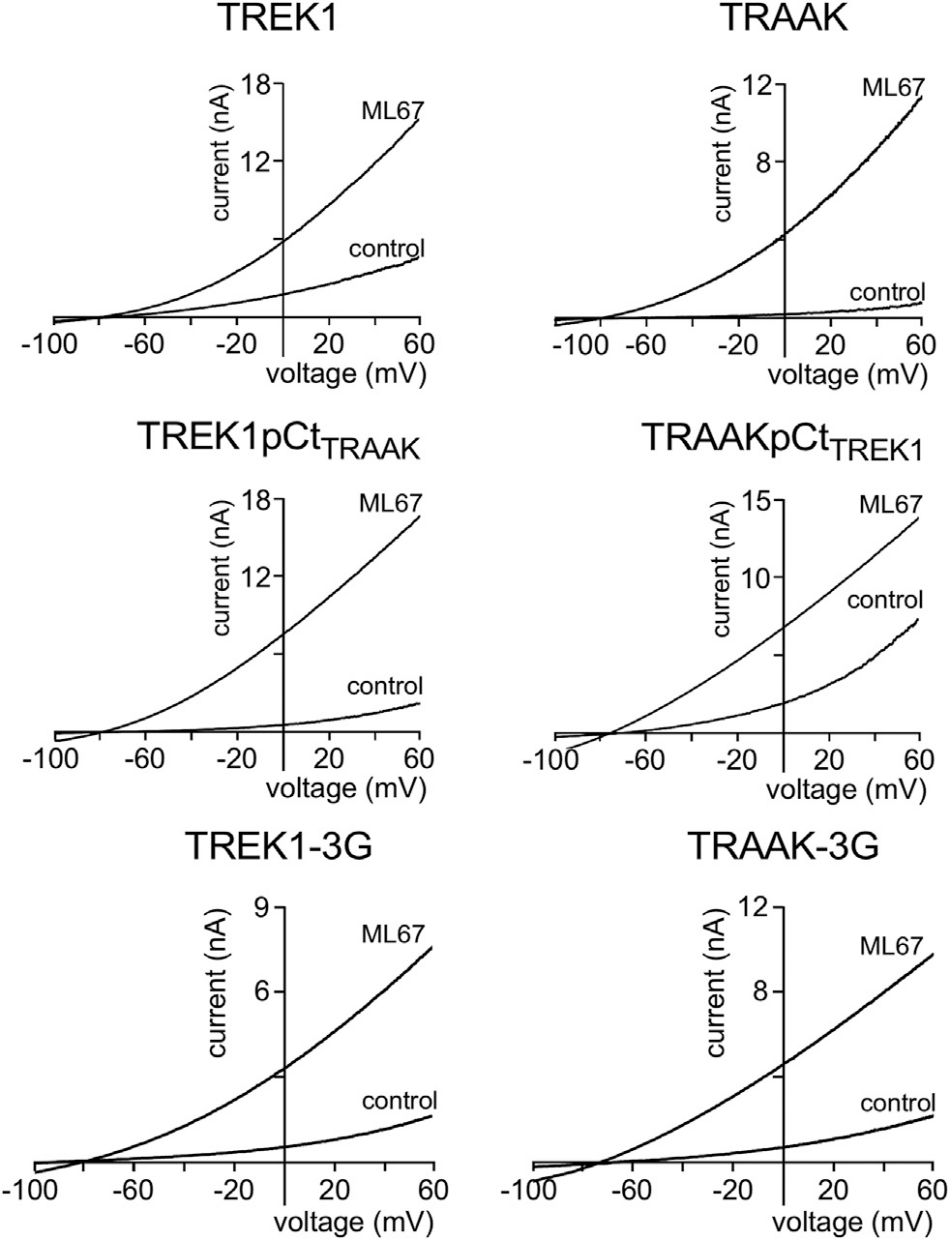
Proximal C-ter domain (pCt) modulates channel sensitivity to drugs. Representative patch-clamp recordings from HEK cells expressing the indicated channels in the presence of ML67 (100 μM). Voltage ramps were applied from −100 to 60 mV from a holding potential of −80mV.

We first studied the sensitivity of TREK1 and TRAAK to the opener ML67 (Figure 2) (Bagriantsev et al., 2013). ML67 stimulates by nearly 5-fold TREK1 (I_drug_/I_control_ = 4.76 ± 0.43, Figures 2, 5A). Under the same conditions, ML67 is even more efficient on TRAAK with a 12-fold increase (I_drug_/I_control_ = 12.38 ± 1.26). Swapping pCt between TREK1 and TRAAK reverses their sensitivity to ML67: TREK1pCt_TRAAK_ becomes more sensitive than TREK1 (I_drug_/I_control_ = 13.97 ± 1.73 *versus* I_drug_/I_control_ = 4.76 ± 0.43), whereas TRAAKpCt_TREK1_ becomes less sensitive than TRAAK (I_drug_/I_control_ = 3.80 ± 0.44 *versus* I_drug_/I_control_ = 12.38 ± 1.26, Figures 2, 5A). Uncoupling pCt from the SF gate, by introducing glycine residues between pCt and M4, also affects the sensitivity of TRAAK to ML67. TRAAK-3G is significantly less sensitive to ML67 than TRAAK (I_drug_/I_control_ = 6.05 ± 0.64 *versus* I_drug_/I_control_ = 12.38 ± 1.26). The 3G-mutation has no effect on TREK1 sensitivity to ML67 (Figures 2, 5A).

**FIGURE 3:**
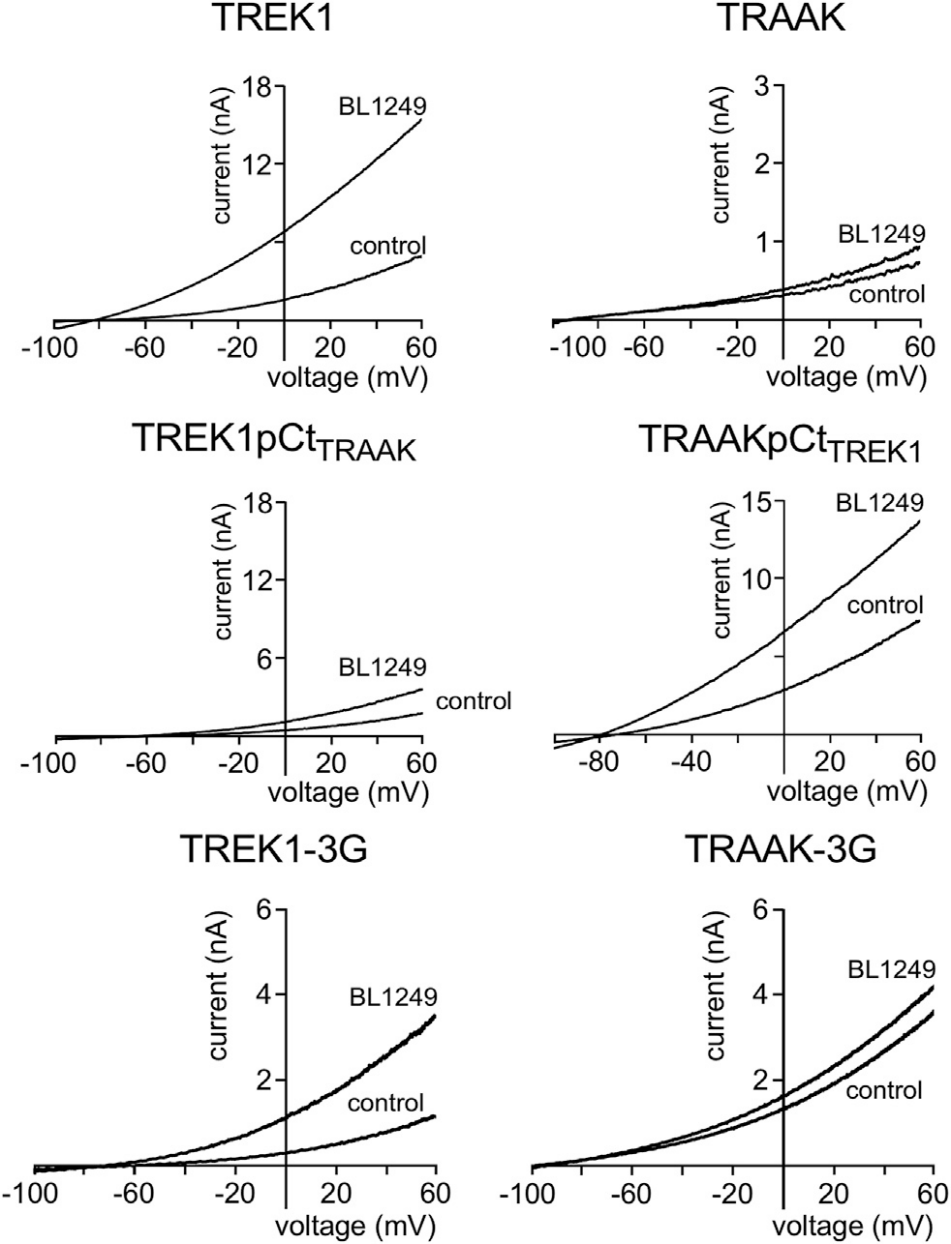
Proximal C-ter domain (pCt) modulates channel sensitivity to drugs. Representative patch-clamp recordings from HEK cells expressing the indicated channels in the presence of BL1249 (10 μM). Voltage ramps were applied from −100 to 60 mV from a holding potential of −80mV.

**FIGURE 4:**
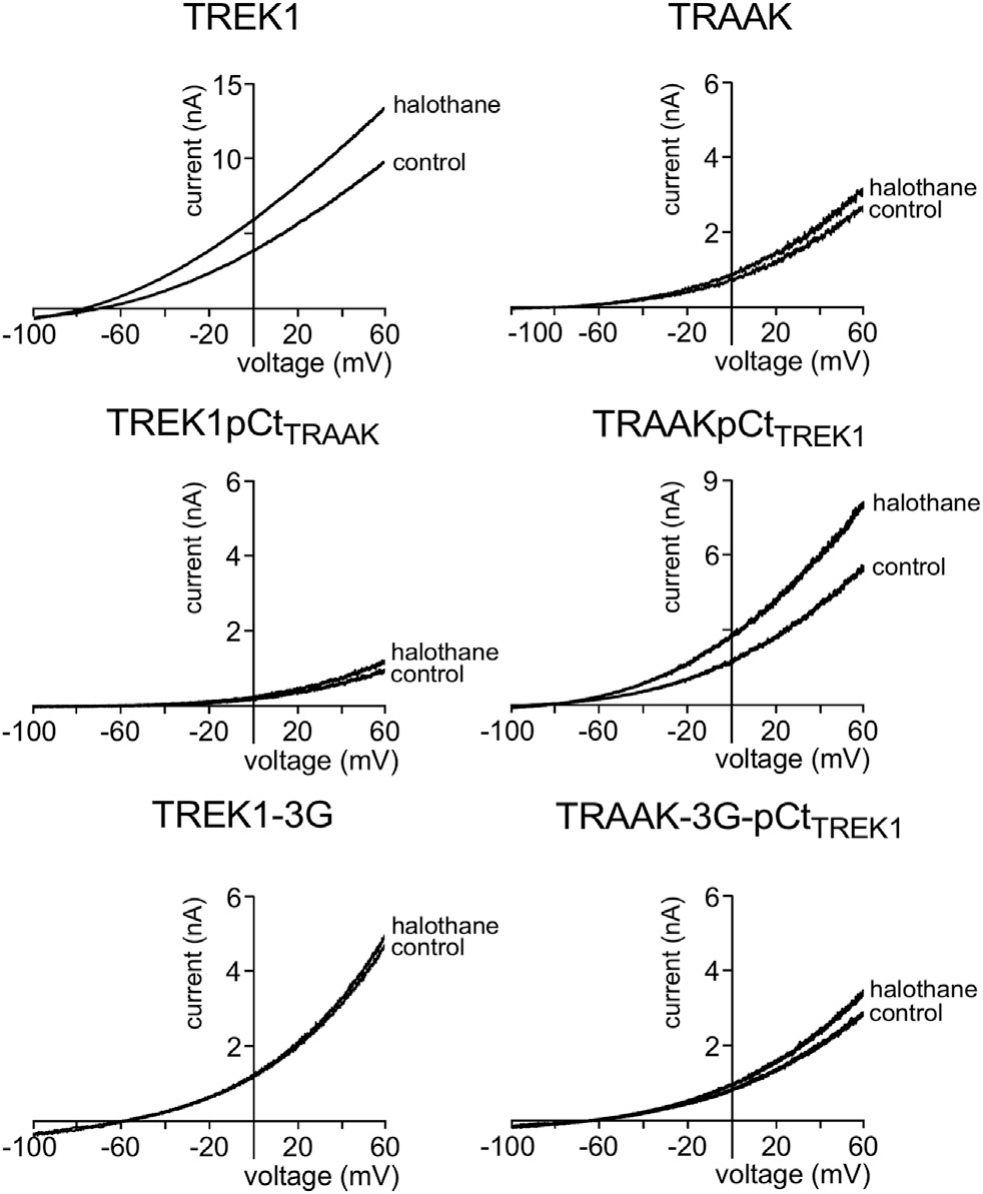
Proximal C-ter domain (pCt) modulates channel sensitivity to drugs. Representative patch-clamp recordings from HEK cells expressing the indicated channels in the presence of halothane (1 mM). Voltage ramps were applied from −100 to 60 mV from a holding potential of −80mV.

Next, we studied the sensitivity of TREK1 and TRAAK to the opener BL1249 (Tertyshnikova et al., 2005). As previously shown, TREK1 is more sensitive to BL1249 than TRAAK (I_drug_/I_control_ = 7.7 ± 1.4 vs I_drug_/I_control_ = 1.16 ± 0.03, Figures 3, 4B) (Pope et al., 2018). The sensitivity of TRAAKpCtTREK1 to BL1249 is significantly higher than that of TRAAK (I_drug_/I_control_ = 2.04 ± 0.31 vs I_drug_/I_control_ = 1.16 ± 0.03), and TREK1pCtTRAAK has a lower sensitivity to BL1249 than TREK1 (I_drug_/I_control_ = 2.49 ± 0.32 vs I_drug_/I_control_ = 7.7 ± 1.4, Figures 3, 4B). Uncoupling pCt/M4 using the 3G-mutation decreases the sensitivity of TREK1 to BL1249 (I_drug_/I_control_ = 3.95 ± 0.32 vs I_drug_/I_control_ = 7.7 ± 1.4) as shown previously by Pope et al. (2018), but had no effect on that of TRAAK (I_drug_/I_control_ = 1.32 ± 0.04 vs I_drug_/I_control_ 1.16 ± 0.03, Figures 3, 4B).

**FIGURE 5:**
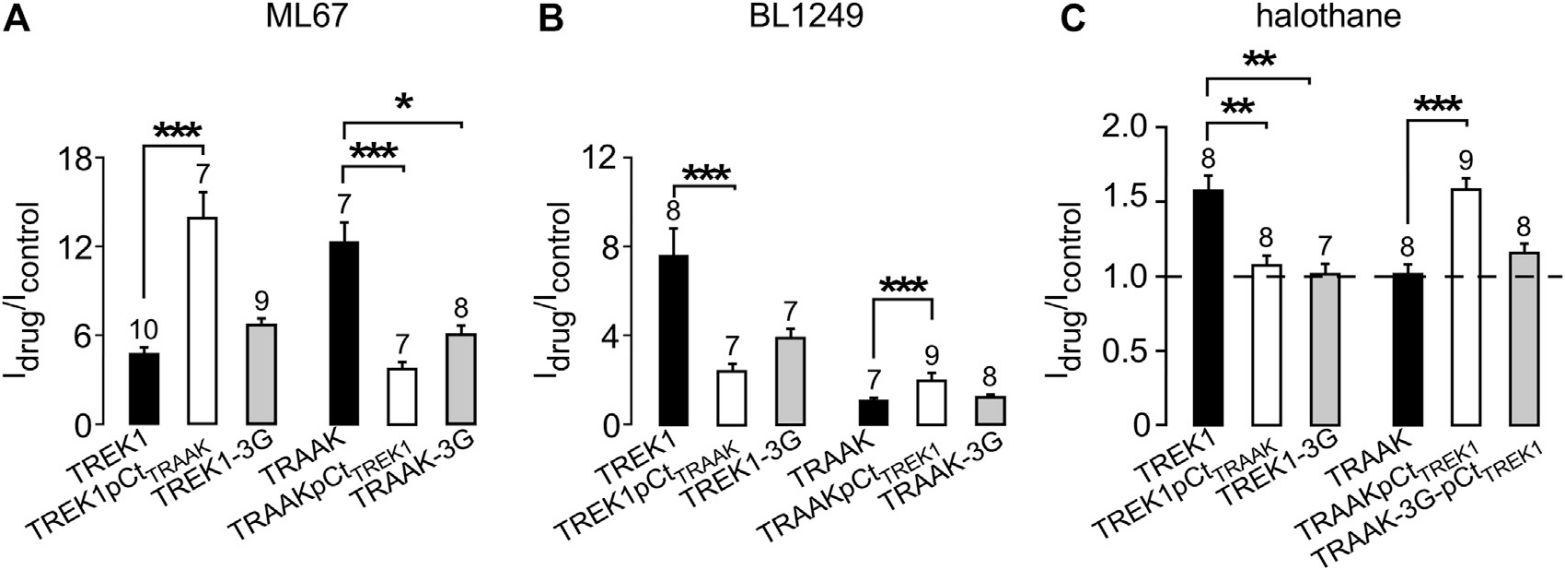
Proximal C-ter domain (pCt) modulates channel sensitivity to drugs. **(A, B, C)** Fraction of thecurrents sensitive to ML67 (100 μM) **(A)**, BL1249 (10 μM) **(B)** and halothane (1 mM), as recorded in Figures 2–4. **(C)**. Data are presented as mean ± SEM, the number of cells is indicated, ∗*p* < 0.05, ∗∗*p* < 0.01, ∗∗∗*p* < 0.001 (Kruskal Wallis).

We also tested the sensitivity of these channels to halothane, a volatile anesthetic. As previously reported, halothane activates TREK1 but not TRAAK (Patel et al., 1999) (Figure 4). TREK1pCt_TRAAK_ has a lower sensitivity to halothane (I_drug_/I_control_ = 1.08 ± 0.07) than TREK1 (Idrug/Icontrol = 1.59 ± 0.08), whereas TRAAKpCt_TREK1_ exhibits sensitivity to halothane (I_drug_/I_control_ = 1.59 ± 0.07, Figures 4, 5C) that is not present in TRAAK (I_drug_/I_control_ = 1.03 ± 0.06). We also evaluated the effect of the 3G-mutation on the sensitivity of TREK1 and TRAAK to halothane. TREK1-3G is no longer sensitive to the drug (I_drug_/I_control_ = 1.03 ± 0.07, Figures 4, 5C). Furthermore, the 3G-mutation also rendered TRAAKpCt_TREK1_ insensitive to halothane as TRAAK (I_drug_/I_contr_ol = 1.16 ± 0.05, Figures 4, 5C), showing that pCt/M4 coupling is necessary for halothane action on TREK1 and TRAAKpCt_TREK1_.

These results show that pCt alters the sensitivity of TREK1 and TRAAK channels to ML67, BL1249 and halothane. The alteration is unique to each opener. Thus, ML67 is more active on TRAAK than on TREK1. The pCt of TRAAK favors activation by ML67 because its introduction into TREK1 increases its activity and its uncoupling to M4 decreases TRAAK sensitivity. Unlike TRAAK pCt, TREK1 pCt has no effect, and its uncoupling to M4 in TREK1-3G does not affect the effect of ML67. Unlike ML67, BL1249 is more active on TREK1 than on TRAAK. TREK1 pCt favors activation by BL1249. Its introduction into TRAAK makes this channel more sensitive to BL1249 whereas its uncoupling to M4 decreases the sensitivity of TREK1 to BL1249. Finally, halothane activates TREK1 but not TRAAK. TREK1 pCt is required for activation by halothane and makes TRAAKpCt_TREK1_ sensitive to this drug. As expected, its uncoupling to M4 in TRAAK-3G-pCt_TREK1_ suppresses this effect. The recent crystal structure of TREK2 interacting with BL1249 showed that this opener binds below the SF to the “fenestration site” at the interface of the M2/M3/M4 domains, likely stabilizing the SF. Like BL1249, ML67 has also been suggested to bind to the “fenestration site” (Schewe et al., 2019), suggesting that both openers act through a similar mechanism. Halothane was suggested to bind to the cytoplasmic C-ter of TREK1 because deletion of the last 48 residues of the C-ter completely abolished channel activation. However, insertion of these same residues into the C-ter of TRAAK did not confer halothane sensitivity (Patel et al., 1999), suggesting that the C-ter influences binding of volatile anaesthetics, but may not be the primary binding site. In full agreement with this, a recent study showed that volatile anaesthetics bind to a pocket formed by the G182 residue located in M2 and the M3-M4 domains, stabilizing the SF gate of the channel (Wague et al., 2020). Together, these data suggest that the effect of these three openers all converges on the same gate that is regulated by pCt. Beside this “distant” allosteric effect of pCt, we cannot exclude that this domain contains residues that may be also directly involved in the effect of the openers, contributing to their binding to the channels or modulating their effect on K^+^ permeation.

### Openers and pCt Have Allosteric Effects on the “up” and “Down” Conformations of TREK1 and TRAAK

**FIGURE 6:**
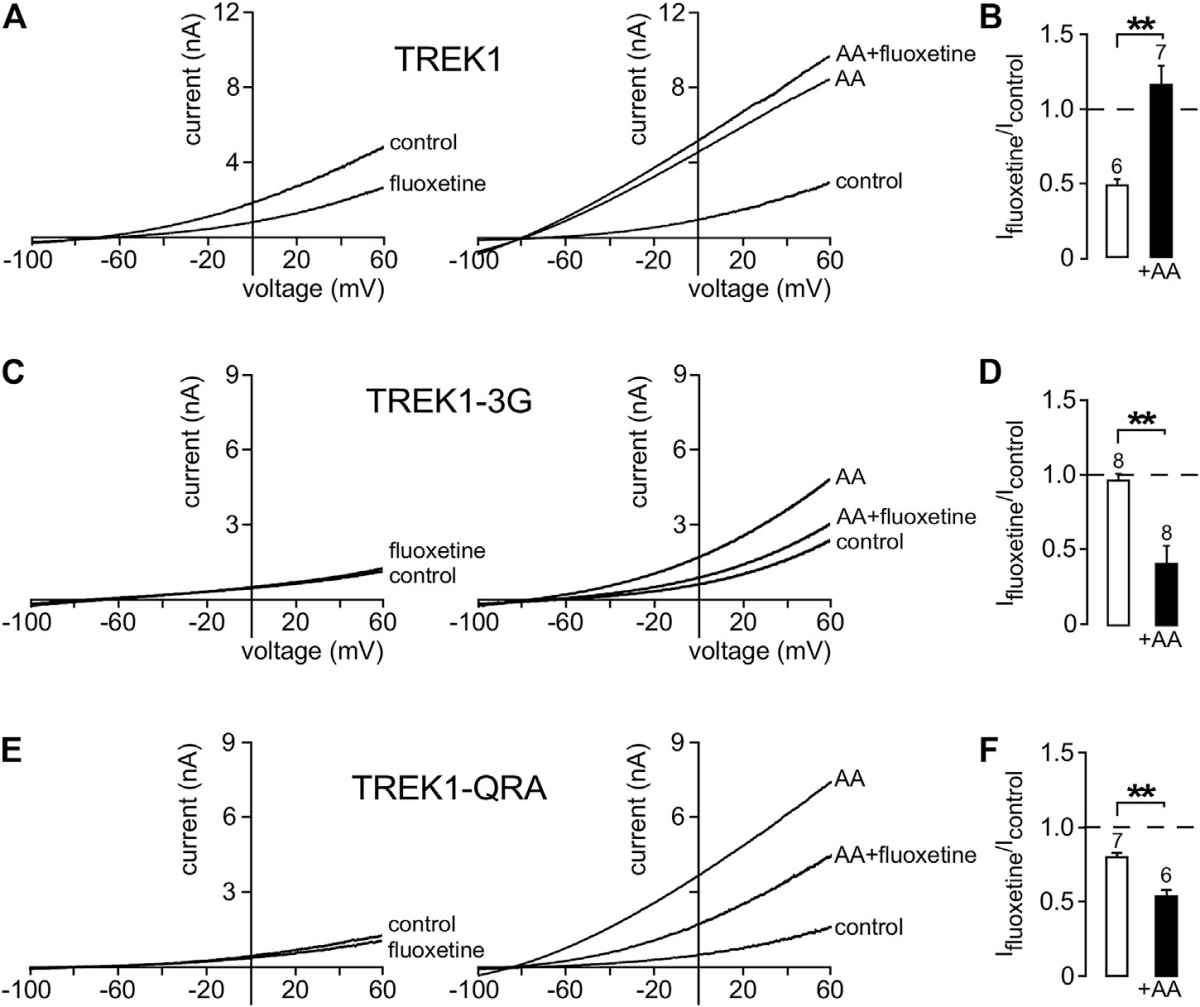
State-dependent inhibitory effect of fluoxetine. **(A, C, E)** Representative whole-cell currents from cells transfected with TREK1 **(A)**, TREK1-3G **(C)** and TREK1-QRA **(E)** channels in the presence or absence of 10 μM fluoxetine on basal currents **(left panel)** and on currents stimulated by AA (10 μM) **(right panel)**. **(B, D, F)** Fraction of fluoxetine-sensitive current in the absence or presence of AA (10 μM). Data are presented as mean ± SEM, the number of cells is indicated, ∗∗*p* < 0.01 (Mann-Whitney test).

In a previous work (Soussia et al., 2018), we showed that pCt modulates the “up” and “down” conformations of TREK1 and TRAAK channels. Since different openers appear to control the same gate in these channels, we next addressed the possibility that openers affect both the “up” and “down” conformations. Using fluoxetine state-dependent binding, we first probed the conformation of TREK1. Under basal condition, TREK1 is in the fluoxetine-sensitive “down” conformation (Figures 6A,B). Unlike TREK1, TREK1-3G behaves like TRAAK that is in a fluoxetine-resistant “up” conformation. Fluoxetine has no effect on the TREK1-3G (I_fluoxetine_/I_control_ = 0.97 ± 0.05). However, upon application of arachidonic acid (AA), TREK1-3G becomes sensitive to fluoxetine (I_fluoxetine_/I_control_ = 0.42 ± 0.11, Figures 6C,D), suggesting that this mutated channel enters the “down” conformation once activated by AA. These results extend our previous ones (Soussia et al., 2018) and show that pCt contributes to the modulation of the conformational state through its coupling to M4. The QRA mutation in the pCt of TREK1 disrupts a potential site of interaction with PIP_2_ (R329, R330, R331) destabilizing the channel conductive state. TREK1-QRA is less active than TREK1. We tested the sensitivity of TREK1-QRA in basal conditions and once activated by AA. Like TRAAK, TREK1-QRA is resistant to fluoxetine under basal conditions, suggesting that this mutated channel is in the “up” state (Figures 6E,F). Upon application of AA, TREK1-QRA reaches a fluoxetine-sensitive “down” state (I_fluoxetine_/I_control_ = 0.52 ± 0.04, Figures 6E,F) confirming that the PIP_2_-binding site in pCt modulates the conformational state of TREK1.

**FIGURE 7:**
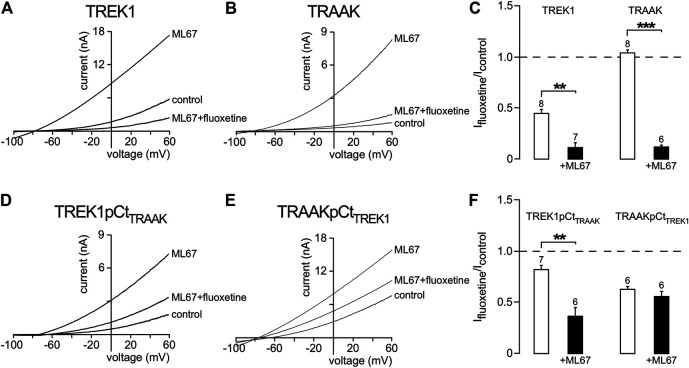
State-dependent inhibitory effect of fluoxetine modulated by ML67. **(A, B, D, E)** Representative patch-clamp recordings from HEK cells expressing wild-type or mutated channels stimulated by ML67 (100 μM) and in the presence of fluoxetine (10 μM). **(C, F)** Fraction of fluoxetine-sensitive current in the absence or presence of ML67 (100 μM). Data are presented as mean ± SEM. ∗∗*p* < 0.01, ∗∗∗ *p* < 0.001, the number of cells is indicated (Mann-Whitney test).

We then asked whether other openers could also induce changes in channel conformation in a pCt-dependent mechanism. Under basal condition, TREK1 is in the fluoxetine-sensitive “down” conformation (Figures 6A,B) whereas TRAAK is in the fluoxetine-resistant “up” conformation (Soussia et al., 2018). Upon ML67 application, TREK1 does not become resistant to fluoxetine (Figures 7A,C). But TRAAK becomes sensitive to fluoxetine once stimulated by ML67 (Figures 7B,C). Similar results were obtained by swapping pCt between TREK1 and TRAAK. Upon application of ML67, TREK1pCt_TRAAK_ switches from resistant to sensitive to fluoxetine (I_fluoxetine_/I_control_ = 0.38 ± 0.08, Figures 7D,F). This action of ML67 is similar to the action of AA on TREK1pCt_TRAAK_ (Soussia et al., 2018). In contrast, TRAAKpCt_TREK1_ behaves like TREK1 and shows no changes in fluoxetine sensitivity upon ML67 application (I_fluoxetine_/I_control_ = 0.56 ± 0.05, Figures 7E,F). Taken together, these results show that ML67 is able to promote a shift of TRAAK from a fluoxetine-resistant state (“up”) to a fluoxetine-sensitive state (“down”) but is not able to change the conformational state of TREK1. Both AA and ML67 activate these channels in a pCt-dependent manner but they do not have the same effect on the conformation of these channels, suggesting that two different allosteric mechanisms occur. One possibility is that the binding of ML67 to these channels stabilizes them in the “down” conformation. This is supported by the observation that the effect of fluoxetine on TREK1 is strongly enhanced in presence of ML67 (Figure 7A) suggesting a stabilization of the channel in the “down” state. It was recently shown that the inhibitory effect of norfluoxetine on TREK2 is neutralized by ML335 and not affected by 2-APB (Proks et al., 2021). Because we have not tested the effect of these openers on TREK1 sensitivity to fluoxetine, we do not know how their binding affects the conformation of this channel.

### pCt Affects the Sensitivity of TREK1 and TRAAK to Extracellular pH

**FIGURE 8:**
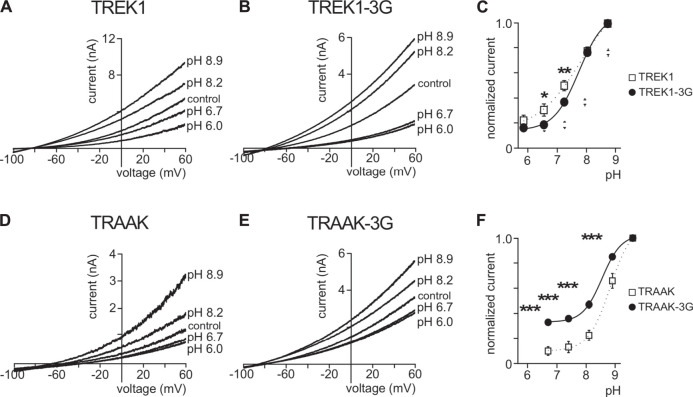
Effect of extracellular pH. **(A, B, D, E)**, Representative patch-clamp recordings from HEK cells expressing wild-type or mutated channels. Voltage ramps were applied from −100 to 60 mV from a holding potential of −80mV. **(C, F)**, Normalized pH response (at 0 mV) for indicated channels. Data are presented as mean ± SEM. ∗*p* < 0.05, ∗∗ *p* < 0.01, ∗∗∗ *p* < 0.001 (Two-way ANOVA).

TREK1 is activated by intracellular acidification through the E306 residue located in the pCt. The mutant TREK1E306A is resistant to intracellular acidification and less active than TREK1 (Honore et al., 2002). TREK1 is inhibited by extracellular acidification and stimulated by extracellular alkalinisation (Sandoz et al., 2009) (Figures 8A,C). Residues involved in this sensitivity are located on the extracellular side of the channel, close to the outer mouth of the pore. Here, we tested the role of pCt on the sensitivity of TREK1 and TRAAK to external pH. We took advantage of the uncoupling of pCt and M4 in TREK1-3G to test a possible allosteric effect of pCt on the regulation by acidification. Inhibition by extracellular acidification was still observed in TREK1-3G in good agreement with the result reported by Bagriantsev et al. (2012), but the normalized remaining current at pH 7.4 and 6.7 was a bit lower than TREK1 (0.19 ± 0.02 vs 0.31 ± 0.05 at pH 6.7; 0.37 ± 0.02 vs 0.50 ± 0.04 at pH 7.4, Figures 8B,C). Similar results were obtained by neutralizing K315 and E306 within the pCt of TREK1 (Woo et al., 2018). The 3G mutation also altered the sensitivity of TRAAK to extracellular pH (Figures 8D–F). The effect is stronger than on TREK1 with a significant fraction of TRAAK-3G being resistant to acidification (Figure 8F). This shows that extracellular pH and pCt trigger structural rearrangements that converge on a same and unique SF gate. However, the allosteric effects are less pronounced than between openers and pCt, suggesting multiple conformational states not equally responsive to extracellular and intracellular stimuli.

## Discussion

Many studies have focused on the structural mechanisms controlling the activity and pharmacology of TREK1, TREK2 and TRAAK channels. According to these studies, TREK/TRAAK channels have two main conformations, “up” and “down” (Brohawn et al., 2014; Lolicato et al., 2014) involving a movement of M4 directly coupled to the SF gate (Miller and Long, 2012; Dong et al., 2015; Zhuo et al., 2016). Our previous work has shown that pCt plays a critical role in channel conformation (Soussia et al., 2018). In basal conditions, TRAAK is in the fluoxetine-resistant “up” conformation whereas TREK1 is in the fluoxetine-sensitive “down” conformation (Soussia et al., 2018). In the same conditions, swapping pCt between these two channels leads TREK1pCtTRAAK to adopt the “up” conformation and TRAAKpCtTREK1 the “down” conformation. Here, we show that pCt also affects the sensitivity of TREK1 and TRAAK to extracellular pH and openers by acting on the SF gate. The recent co-crystallisation of TREK2 with a brominated BL1249 has shown that this drug binds to the “fenestration site” (Schewe et al., 2019). This site is located below the P2 pore helix and exposed by the “down” movement of M4 (Brohawn et al., 2012; Miller and Long, 2012; Brohawn et al., 2014; Lolicato et al., 2014). Like BL1249, ML67 also binds to the “fenestration site” (Bagriantsev et al., 2013; Schewe et al., 2019). Since this “fenestration site” is only accessible in the “down” state, we expected TREK1 to be sensitive to BL1249 and ML67, but not TRAAK. Surprisingly, TRAAK is sensitive to both openers like TREK1. However, TRAAK is more sensitive to ML67 than BL1249. Given that pCt affects channel conformation (Soussia et al., 2018), we wondered whether pCt could also affect the sensitivity of TREK1 and TRAAK channels to openers. Replacing TREK1 pCt by that of TRAAK increased the sensitivity of TREK1pCt_TRAAK_ to ML67 and decreased that to BL1249. In contrast, TRAAKpCt_TREK1_ displayed a lower sensitivity to ML67 and a higher sensitivity to BL1249. Similar effects were observed with the 3G-mutation, which decouples pCt from the SF. These data indicate that pCt affects allosterically binding of these openers to the “fenestration site”.

A recent study on volatile anaesthetics, which activate TREK1 (Patel et al., 1999) but not TRAAK, identified a binding site with a glycine as a key element at the M2-M3-M4 interface (Wague et al., 2020). Interestingly, this binding site corresponds to the “fenestration site”, showing that different types of openers bind to this site. Swapping pCt between TREK1 and TRAAK or disrupting pCt/SF coupling altered the sensitivity to halothane. These findings confirm the allosteric effects of pCt on SF gate *via* the “fenestration site”. However, key questions remain unresolved: how does pCt affect drug binding to the “fenestration site”? And how does the binding of molecules to this “fenestration site” affect the SF state? A recent structural study on the TREK2/BL1249 complex gave new insights. The negatively charged tetrazole moiety of BL1249 promotes the SF conductive state by changing the coordination environment of K^+^ at position S1 and S4, increasing ion permeation (Schewe et al., 2019). ML67 contains a negatively charged moiety like BL1249 (Bagriantsev et al., 2013; Schewe et al., 2019), suggesting that BL1249 and ML67, and possibly halothane, may stabilize the SF gate in a conductive state through a similar mechanism. External pH, which regulates TREK1, may also affect the SF gate by modulating ion permeation. Comparing the structures of the TREK-related TASK2 channel at pH 6.5 and pH 8.5 revealed that external acidification induces a distortion of the SF at the S0 and S1 positions that prevents K^+^ to occupy these positions (Li et al., 2020). TREK1 and TASK2 are similarly sensitive to external pH, suggesting that pCt in TREK1 may also affect the sensitivity to external pH in an allosteric manner. Consistent with this, we found that disrupting pCt/SF coupling with the 3G-mutation changed pH-sensitivity of TREK1 and TRAAK. Not only openers bind to this “fenestration site” but also inhibitors, including fluoxetine and norfluoxetine (Miller and Long, 2012; Dong et al., 2015; Zhuo et al., 2016), indicating that the “fenestration site” is a dual-action site for its ligands. Identifying the precise molecular mechanism by which ligands influence the conformation site of the SF gate is key to the development of novel modulators of TREK/TRAAK channels. It has to be noted that the “fenestration site” is composed of elements from both subunits of the active dimers (Natale et al., 2021), suggesting that homo and heterodimers could be specifically modulated (Blin et al., 2016; Lengyel et al., 2016; Levitz et al., 2016), enhancing the interest in developing drugs specifically targeting this region.

In summary, the allosteric effect of pCt on the stimulatory action of ML67, BL1249, AA and halothane shows that these stimuli converge to the same SF gate. This gate is the same as the gate controlled by extracellular pH. Although the openers act through the same SF gate, they do not affect the conformational states of these channels in the same way. Previously, we showed that AA promotes the shift from the “up” to the “down” conformation of TREK1 and from the “down” to the “up” conformation of TRAAK (Soussia et al., 2018). Here, we showed that the 3G-mutation, which uncouples pCt from SF, changed the conformational state of TREK1 as expected. Surprisingly, and in contrast to AA, ML67 only promotes the transition from the “up” to the “down” conformation of TRAAK under basal conditions. TREK1 remains in the “down” conformation upon ML67 application. Taken collectively, these results provide further evidence that the crystallography identified “up” and “down” conformations do not correspond to open or closed states of these channels.

## Data Availability

The raw data supporting the conclusions of this article will be made available by the authors, without undue reservation.
