# Corrigendum: Convergence of Multiple Stimuli to a Single Gate in TREK1 and TRAAK Potassium Channels

**DOI:** 10.3389/fphar.2021.795920

**Published:** 2021-11-17

**Authors:** Frank S. Choveau, Ismail Ben Soussia, Delphine Bichet, Franck C. Chatelain, Sylvain Feliciangeli, Florian Lesage

**Affiliations:** Université Côte D’Azur, INSERM, CNRS, Institut de Pharmacologie Moléculaire et Cellulaire, LabEx ICST, Valbonne, France

**Keywords:** two-pore domain potassium channels, pore gating, openers, allostery, extracellular pH

In the original article, there was an error. We forgot to mention the use of the TREK1-3G construct in previous studies reporting similar results. Two corrections have been made. The first correction has been made to section **Results,** sub-section **Openers and pCt Have Allosteric Effects on the Channel Activity of TREK1 and TRAAK**, paragraph 3:

“Next, we studied the sensitivity of TREK1 and TRAAK to the opener BL1249 (Tertyshnikova et al., 2005). As previously shown, TREK1 is more sensitive to BL1249 than TRAAK (I_drug_/I_control_ = 7.7 ± 1.4 vs I_drug_/I_control_ = 1.16 ± 0.03, Figures 3, 4B) (Pope et al., 2018). The sensitivity of TRAAKpCtTREK1 to BL1249 is significantly higher than that of TRAAK (I_drug_/I_control_ = 2.04 ± 0.31 vs I_drug_/I_control_ = 1.16 ± 0.03), and TREK1pCtTRAAK has a lower sensitivity to BL1249 than TREK1 (I_drug_/I_control_ = 2.49 ± 0.32 vs I_drug_/I_control_ = 7.7 ± 1.4, Figures 3, 4B). Uncoupling pCt/M4 using the 3G-mutation decreases the sensitivity of TREK1 to BL1249 (I_drug_/I_control_ = 3.95 ± 0.32 vs I_drug_/I_control_ = 7.7 ± 1.4) as shown previously by Pope et al. (2018), but had no effect on that of TRAAK (I_drug_/I_control_ = 1.32 ± 0.04 vs I_drug_/I_control_ 1.16 ± 0.03, Figures 3, 4B).”

The second correction has been made to section **Results**, sub-section **pCt Affects the Sensitivity of TREK1 and TRAAK to Extracellular pH**, paragraph 1:

“TREK1 is activated by intracellular acidification through the E306 residue located in the pCt. The mutant TREK1E306A is resistant to intracellular acidification and less active than TREK1 (Honore et al., 2002). TREK1 is inhibited by extracellular acidification and stimulated by extracellular alkalinisation (Sandoz et al., 2009) (Figures 8A,C). Residues involved in this sensitivity are located on the extracellular side of the channel, close to the outer mouth of the pore. Here, we tested the role of pCt on the sensitivity of TREK1 and TRAAK to external pH. We took advantage of the uncoupling of pCt and M4 in TREK1-3G to test a possible allosteric effect of pCt on the regulation by acidification. Inhibition by extracellular acidification was still observed in TREK1-3G in good agreement with the result reported by Bagriantsev et al. (2012), but the normalized remaining current at pH 7.4 and 6.7 was a bit lower than TREK1 (0.19 ± 0.02 vs 0.31 ± 0.05 at pH 6.7; 0.37 ± 0.02 vs 0.50 ± 0.04 at pH 7.4, Figures 8B,C). Similar results were obtained by neutralizing K315 and E306 within the pCt of TREK1 (Woo et al., 2018). The 3G mutation also altered the sensitivity of TRAAK to extracellular pH (Figures 8D–F). The effect is stronger than on TREK1 with a significant fraction of TRAAK-3G being resistant to acidification (Figure 8F). This shows that extracellular pH and pCt trigger structural rearrangements that converge on a same and unique SF gate. However, the allosteric effects are less pronounced than between openers and pCt, suggesting multiple conformational states not equally responsive to extracellular and intracellular stimuli.”

The authors apologize for this error and state that this does not change the scientific conclusions of the article in any way. The original article has been updated.

